# Olive leaf extract and butylated hydroxytoluene as antioxidants in refined versus whole wheat flour crackers: a physico‐chemical characterization

**DOI:** 10.1002/jsfa.70248

**Published:** 2025-10-25

**Authors:** Ottavia Parenti, Maria Paciulli, Eleonora Carini, Francesca Bot, Emma Chiavaro

**Affiliations:** ^1^ Department of Food and Drug University of Parma Parma Italy

**Keywords:** cereal snacks, plant‐based antioxidants, olive oil by‐products, unrefined wheat flour, ^1^H NMR relaxometry

## Abstract

**BACKGROUND:**

This study investigated the physico‐chemical properties of four cracker samples formulated with refined (RF) or whole wheat flour (WF), and enriched with olive leaf extract in either free form (OLE) or encapsulated form (MCR), as well as with butylated hydroxytoluene (BHT) antioxidant, in comparison with non‐enriched control samples (CTR).

**RESULTS:**

The MCR sample showed the highest total antioxidant capacity. Free OLE showed an intermediate value among all the tested samples and OLE reduced the peroxide value significantly in comparison with CTR. Encapsulated OLE was similar to the other formulations. The WF crackers showed lower moisture content, softer texture, and a lower impact of the natural antioxidant on color the parameters than the RF samples. The antioxidant type displayed little impact on the physical properties of crackers, with MCR showing the highest moisture content and texture consistency, and free OLE was comparable to CTR and BHT. Although free OLE revealed the greatest impact on the crackers' color, encapsulation significantly reduced this effect. Significant changes in proton nuclear magnetic resonance (^1^H NMR) parameters were mainly associated with the higher fiber and lower gluten content of WF in comparison with RF; the type of antioxidant used affected the size of its impact.

**CONCLUSIONS:**

The results revealed the potential of encapsulated OLE as a natural antioxidant for the development of clean label snacks. © 2025 The Author(s). *Journal of the Science of Food and Agriculture* published by John Wiley & Sons Ltd on behalf of Society of Chemical Industry.

## INTRODUCTION

Crackers are popular snacks. They are defined as thin, dry, crisp baked products, usually made with wheat flour, containing leavening agents and low levels of sugar and moisture.^1^, ^2^, ^3^ However, their low water activity (*a*_w_) (0.2–0.3) and the presence of fats make crackers susceptible to chemical spoilage or rancidity, causing lipid oxidation. A negative impact on the sensory profile (‘off’ odors and flavors) and on nutritional value, as well as a reduction of their shelf‐life can occur.^4^ To prevent and delay lipid oxidation in such products, antioxidants are generally added to food formulations. The most widely used synthetic antioxidants in bakery products are butylated hydroxyanisole (BHA), and butylated hydroxytoluene (BHT).^4^, ^5^ However, health issues due to the long‐term intake of synthetic antioxidants,^5^, ^6^ as well as poor consumer acceptance of E‐numbers additives,^7^ are driving the replacement of synthetic antioxidants with plant‐derived alternatives.^5^, ^6^ Plant‐derived antioxidants, such as polyphenols,^8^ have the advantage that they are ‘generally recognized as safe’ (GRAS), although they are subject to the same strict safety standards applied to synthetic additives.^5^, ^6^, ^9^

Olive (*Olea europaea* L., Oleaceae) is one of the most extensively cultivated crops in the world, with Mediterranean countries accounting for 98% of the world's olive production.^10^, ^11^ Olive fruits are mainly used for olive oil extraction, a process that produces many by‐products, including olive leaves (OL), olive stones, olive oil pomace, and olive oil waste water, all of which represent valuable sources of antioxidants.^11^, ^12^ Olive leaves, which represent about 100 g kg^−1^ of the weight of olives collected for oil extraction,^13^ include a mixture of leaves, small twigs and branches containing a considerable number of phenolic compounds.^14^ Several studies have highlighted the potential of phenolic compounds extracted from olive oil by‐products in different food matrices, including baked goods.^9^, ^12^ However, phenolic compounds are quite reactive and sensitive to the factors to which food products are exposed during processing and storage as temperature, pH, and light.^15^ Microencapsulation represents an interesting and effective technological strategy to protect phenolic‐rich extracts,^12^, ^16^ as has been shown in low‐moisture bakery products formulated with olive leaf extract (OLE) as an antioxidant.^17^, ^18^, ^19^ These studies found that the enrichment of wheat flour breadsticks, gluten free breadsticks, and biscuits with different amounts of OLE (400 mg kg^−1^ of flour, 500 and 1000 mg kg^−1^ of flour, 500 μg GAE g^−1^) improved antioxidant activity, extended product shelf life significantly, and showed a tendency to improve the sensory profile.^17^, ^18^, ^19^

In addition to growing attention to clean‐label and sustainability issues, interest in the use of whole grains has increased in recent years.^20^ Observational and interventional studies have shown that a higher consumption of whole grain is associated with a lower incidence of cardiovascular diseases, type 2 diabetes, and some cancers.^20^, ^21^ The substitution of refined wheat flours (RF) with whole wheat flour (WF) has been investigated for its effects on the technological properties of bread.^20^, ^22^ In contrast, little information is available on the use of WF to produce crackers,^1^, ^2^, ^23^, ^24^, ^25^, ^26^ and even less on the comparative technological performance of WF in comparison with RF.^1^, ^23^

Considering the limited literature on the potential of OLE as a substitute for a synthetic antioxidant in cereal‐based snacks, and the increasing interest in the use of WF, the present study investigated the effect of the addition of OLE in free and encapsulated forms on the physico‐chemical properties of RF and WF crackers. Crackers enriched with OLE were compared with those with an added synthetic antioxidant (BHT) and with a control sample without antioxidant.

## MATERIALS AND METHODS

### Materials

Wheat (*Triticum aestivum* L.) mixture from the varieties *Gentil Rosso, Andriolo, Inallettabile, Autonomia B* was grown in Montespertoli (Florence, Italy) in the 2021–2022 growing season. Wheat kernels were processed at Molino Paciscopi (Montespertoli, Florence, Italy) in a single batch using a roller mill to produce two flour types based on the Italian classification.^27^, ^28^ Refined wheat flour had an extraction rate of 75 g 100 g⁻¹ dry kernels and a maximum ash content of 0.65 g 100 g⁻¹, whereas WF had an extraction rate of 98 g 100 g⁻¹ dry kernels and a maximum ash content of 1.3–1.7 g 100 g⁻¹.

Fresh brewer's yeast (AB Mauri Italy S.p.A., Casteggio, Pavia, Italy), salt (Italkali S.p.A., Petralia Soprana, Palermo, Italy), and water (Nocera Umbra Fonti Storiche S.p.A., Nocera Umbra, Perugia, Italy) were purchased from a local supermarket (Conad, Parma, Italy). Extra virgin olive oil was purchased from Oleificio Coppini Angelo S.p.A. (Terni, Italy). Butylated hydroxytoluene (Sigma‐Aldrich, St Louis, MO, USA) was provided as a powder, added to a known quantity of water and then included in the dough. Olive leaf extract in the form of powder (oleuropein content ≥400 g kg^−1^ of extract) was supplied by Panakeia (Teramo, Italy). Specifically, the powder was obtained using the following steps: preparation and cleaning of olive leaves, double extraction of the material using ethanol for 3 h, concentration of the extract at a temperature below 60 °C, and centrifugation. The resulting clear liquid was separated from the residual ethanol, purified with a resin column, filtered, and spray dried to obtain a powder, which was stored under vacuum, at −18 °C, and in the dark to prevent its degradation. The safety of the olive leaf extract conformed to the chemical and microbiological parameters reported in Table 1. Pea protein concentrate powder (800 g kg^−1^ proteins) was a commercial product. All reagents used in the study were of analytical grade and sourced from Sigma‐Aldrich unless explicitly specified.

**Table 1:** Chemical and microbiological parameters of OLE

	Limits
Chemical parameters	
Heavy metals	≤ 10 ppm
Lead	< 3 ppm
Cadmium	< 1 ppm
Mercury	< 0.1 ppm
Microbiological parameters	
Total bacterial count	≤ 1000 cfu/g
Yeast and molds	≤ 100 cfu/g
*E. coli*	Absent/1 g
Salmonella	Absent/100 g
Aflatoxins	
Total aflatoxins (B1, B2, G1, G2)	≤ 10 ppm
Aflatoxin B1	≤ 5 ppb
PAH (Reg. 1993/2015/EU)	
PAH (benzo[a]pyrene, benzo[a]anthracene, benzo[a]fluoranthene, chrysene)	≤ 50 ppb
Benzo[a]pyrene	≤ 5 ppb
Residual solvents	Conform to P.E. 8.0
Pesticides	Conform to Reg. 2005/396/CE
Ethylene oxide	Conform to Reg. 2005/396/CE

Abbreviation: OLE, olive leaf extract.

### Formulation of cracker samples

The following eight cracker samples were produced:

- RF‐CTR – crackers with RF and without the addition of antioxidant;
- WF‐CTR – crackers with WF and without the addition of antioxidant;
- RF‐BHT – crackers with RF and BHT;
- WF‐BHT – crackers with WF and BHT;
- RF‐OLE – crackers with RF and OLE in a free form;
- WF‐OLE – crackers with WF and OLE in a free form;
- RF‐MCR – crackers with RF and OLE in an encapsulated form;
- WF‐MCR – crackers with WF and OLE in an encapsulated form.

Crackers were formulated as described by Manzocco *et al*.,^29^ with some modifications, as reported in Table 2. The crackers enriched with BHT (i.e., RF‐BHT and WF‐BHT) were prepared using the CTR sample formulation with the addition of 200 mg of BHT kg^−1^ of oil, corresponding to 48 mg BHT kg^−1^ of flour, which complies with the maximum level permitted by European Regulation No. 1129/2011.^30^ In the crackers formulated with OLE, the amount of OLE was selected according to preliminary sensory triangle tests, initially using 500 μg GAE g^−1^ of dough (810 μg GAE g^−1^ of flour), as reported by Paciulli *et al*.,^19^ and exploring a wider range, up to 5814 mg OLE kg^−1^ flour. Crackers with 810 mg of OLE kg^−1^ of flour were not significantly different from the control, whereas the higher OLE levels (i.e., 3400 and 5814 mg OLE kg^−1^ flour) were perceived as bitter.

**Table 2:** Type and amount of ingredients for crackers' formulation

Sample	Wheat flour (g)	Water (g)	Extra virgin olive oil (g)	Salt (g)	Brewer's yeast (g)	Antioxidant (mg)
RF‐CTR	1000	360	240	14	8	–
WF‐CTR	1000	360	240	14	8	–
RF‐BHT	1000	360	240	14	8	48
WF‐BHT	1000	360	240	14	8	48
RF‐OLE	1000	360	240	14	8	1820
WF‐OLE	1000	360	240	14	8	1820
RF‐MCR	1000	360	240	14	8	5460
WF‐MCR	1000	360	240	14	8	5460

Abbreviations: RF‐CTR, refined wheat flour cracker without the addition of antioxidant; WF‐CTR, whole wheat flour cracker without the addition of antioxidant; RF‐BHT, refined wheat flour cracker with the addition of BHT; WF‐BHT, whole wheat flour cracker with the addition of BHT; RF‐OLE, refined wheat flour cracker with the addition of OLE in a free form; WF‐OLE, whole wheat flour cracker with the addition of OLE in a free form; RF‐MCR, refined wheat flour cracker with the addition of encapsulated OLE; WF‐MCR, whole wheat flour cracker with the addition of encapsulated OLE.

A second sensory test was conducted to evaluate OLE concentrations below 3400 mg kg⁻¹ flour and identified 1820 mg kg⁻¹ flour as the appropriate concentration. This level corresponded to the absolute threshold of taste perception according to the Method of Limits.^31^

The OLE powder was first dissolved in all the water used to prepare the cracker dough, and stirred for 10 min at 600 rpm to obtain a homogeneous solution characterized by a light brown color. The solution was then added to the cracker dough for RF‐OLE and WF‐OLE. The samples containing the extract in the encapsulated form were prepared as described by Hidalgo *et al*.^32^ with some modifications. Specifically, the OLE was added in a ratio of 2:1 to 1 g L^−1^ pea protein isolate water solution, previously thermally treated at 90 °C for 30 min. The mixture was freeze‐dried using a freeze‐dryer (LIO5P DIGITAL 5 PASCAL, Levanchimica S.r.l., Bari, Italy), then stored at −18 °C in the dark until use. To maintain the same concentration of OLE, 5460 mg of MCR OLE per kg of flour (comprising 1820 mg of OLE and 3640 mg of pea proteins) was dissolved in water and incorporated into the dough.

All the ingredients were mixed in a professional mixer (Kitchen‐Aid Deluxe, St Joseph, MI, USA) with a dough hook (K45DH) at the lowest speed (speed 1) for 3 min. The dough was allowed to rest for 30 min at 25 °C in a covered bowl, then it was laminated to 1 mm thickness using the mixer (Kitchen‐Aid Deluxe) equipped with a dough sheeter tool and cut into 4.0 cm × 6.0 cm rectangles. All the samples were pierced on the surface to assure uniform cooking and avoid the formation of air bubbles. Samples were cooked in an electric oven (Ixelium, Mod. AKZM 756/IXL, Whirlpool Italia S.r.l., Italy) at 200 °C in static mode for 4.5 min (RF samples) or 5.5 min (WF samples) for each side and cooled down at room temperature in the dark for 1 h before the analyses. The optimal cooking times for RF and WF cracker samples were determined in preliminary trials in which different cooking times were tested, and cracker color, texture and sensory properties were assessed. Three batches for each cracker formulation were prepared and analyzed.

### Physico‐chemical properties of crackers

#### Determination of peroxide value

Lipids were extracted from the crackers, previously milled with a grinder (Osterizer, Hattersheim a. Main, Germany), using diethyl ether in a sample to solvent ratio of 1:10 (w/v) by using a laboratory shaker (600 rpm) under ambient conditions for 60 min. After filtration of the solid using a paper filter, the emulsion was transferred into a round‐bottomed flask, which had been weighed previously on an analytical balance. The lipids were separated by evaporating the solvent under vacuum condition onto the rotary evaporator (Steroglass STRIKE 300, Perugia, Italy) at 30 °C.

The extracted lipids were weighed and used immediately to measure the peroxide value in accordance with European Regulation n. 2568/91.^33^ In detail, 10 mL of dichloromethane, 15 mL of glacial acetic acid, and 1 mL of saturated potassium iodide solution were sequentially added to the extracted fat. The solution was shaken manually for 1 min, kept in the dark for 5 min under ambient conditions, and then 75 mL of distilled water and 3 mL of starch indicator were added. The titration was performed using a sodium thiosulfate solution (0.01  n_eq_ M) and the peroxide value was calculated with the following formula:

(1) $$\text{peroxide value}=\frac{\left(V\times T\right)}{m}\times 1000$$

where *V* and *T* are respectively the volume (mL) and the concentration (n_eq_ M) of the sodium thiosulfate solution used and *m* is the weight (g) of the extracted lipids. The results are expressed as milli‐equivalents of active oxygen per kg of oil (meqO_2_ ${\text{kg}}_{{\text{oil}}^{–1}}$).

Three replicates of lipid extraction were performed for each of the three batches, yielding a total of 3 × 3 = 9 peroxide value measurements per formulation.

#### Total antioxidant capacity

The antioxidant compounds of the crackers were extracted in a 50 mL flask by adding 30 mL of methanol‐distilled water solution (70:30 v/v) to 9 g of milled crackers (Osterizer). The next steps were stirring at 600 rpm for 120 min at room temperature, centrifugation of 5 mL samples in Eppendorf tubes (15 312 × *g*, 10 min, 24 °C) to separate the solid and liquid phases (containing the extracted antioxidants), and filtration through 0.2 μm filters to remove turbidity. Two extractions were performed for each production batch, for a total of 2 extraction replicates × 3 batches = 6 extracts for each formulation.

The total antioxidant capacity of crackers was determined by a 1,1‐diphenyl‐2‐picrylhydrazil (DPPH) assay, following a method described by Brand‐Williams *et al*.^34^ with minimal modifications according to Paciulli *et al*.^19^ The term ‘total antioxidant activity’ was used to indicate the overall effectiveness of the samples in neutralizing free radicals within the context of the DPPH assay, in terms of DPPH scavenging capacity.

The DPPH reagent (1.5 mL, 0.05% w/v = 0.05 g L^−1^ DPPH in methanol) and 500 μL sample extract were mixed, covered with parafilm, and kept in the dark at room temperature for 30 min. A blank sample was prepared with the addition of 50 μL of a methanol‐distilled water 70:30 (v/v) solution instead of the sample extract. Then, the absorbance at 517 nm was measured. The optical density was compared with a standard curve prepared from a 0.1 mg mL⁻¹ Trolox stock solution, diluted to 0.005–0.05 mg mL⁻¹ in a solution of methanol: water (70:30). The results were expressed as Trolox equivalent antioxidant capacity per g of dry matter (TEAC g dry matter^−1^).

The DPPH radical scavenging activity was determined using the following equation:

(2) $$I\%=\left[\left(A0-A1/A0\right)\times 100\right]$$

where A0 is the absorbance of the blank and A1 is the absorbance of the samples.

For each formulation and for each batch of production, the DPPH assay was performed in two replicates. The total number of samples analyzed was the following: 2 replicates of extraction × 2 replicates of analyses × 3 batches = 12 measurements for each formulation.

#### Moisture content

The moisture content of crackers, previously milled with a grinder (Osterizer), was measured according to the gravimetric method AACC 44–15.02.^35^ Three replicates of analysis were performed for each formulation and each production batch for a total of 3 analysis replicates × 3 batches = nine measurements for each formulation.

#### Texture

A penetration test was conducted to evaluate the texture of the samples, using the TA.XT2i texture analyzer instrument equipped with a 30 kg load cell (Stable Micro Systems, Godalming, UK), previously calibrated. For the test, each sample was positioned on the perforated steel surface of the texture analyzer, presenting a 1 cm diameter cylindrical hole. A cylindrical probe, measuring 3 mm in diameter (code SMSP/3), was then employed to penetrate each sample perpendicularly.

The analysis parameters were as follows: pre‐ and post‐test speed, 10.00 mm s⁻¹; test speed, 2.00 mm s⁻¹; probe travel distance, 2500 mm; and trigger force, 0.100 N. The results were processed using Exponent software (version 6.1.16.0), evaluating (i) the maximum penetration force (F, N) and (ii) the area under the force–time curve (area, N s), representing the energy required to penetrate the sample. Each formulation was tested in ten replicates per production batch, with three batches per formulation, yielding a total of 30 measurements.

#### Color analysis

The CIELab color parameters L*, a*, and b* of crackers were measured with the Minolta colorimeter (CM 2600d; Minolta Co., Osaka, Japan) equipped with a standard D65 illuminant.^36^ Spectramagic software (version 3.40) was used for data analysis. The color difference (*Δ*E) was calculated to test the effect of the antioxidant on the two clusters of RF and WF samples, considering RF‐CTR and WF‐CTR as the respective reference standards. *Δ*E was calculated by applying the following formula:

(3) $$\mathrm{\Delta E}=\sqrt[{2}]{{\left(\Delta L^{*}\right)}^{2}+{\left(\Delta a^{*}\right)}^{2}+{\left(\Delta b^{*}\right)}^{2}}$$

Ten analyses were performed for each production batch of each cracker sample, resulting in a total of 30 measurements per formulation (10 replicates × 3 batches).

### Proton molecular mobility and dynamics of cracker doughs

Proton molecular mobility and dynamics were investigated with a low‐resolution (20 MHz) proton nuclear magnetic resonance (^1^H NMR) spectrometer (the MiniSpec; Bruker Biospin, Milan, Italy) operating at 25.0 ± 0.1 °C. Proton (¹H) free induction decay (FID) and *T*₂ Carr–Purcell–Meiboom–Gill (CPMG) experiments were performed. The FID experiments enabled detection of very short relaxation times (10–500 μs), corresponding to the less mobile protons in solid‐like components and to water protons tightly associated with solids. In contrast, the more mobile protons (0.1–1000 ms) were detected using the CPMG pulse sequence. For this analysis, cracker doughs were prepared with the same ingredients and processing conditions used for cracker production (Table 2), except that yeast was omitted to prevent air bubble formation. Immediately after kneading, five samples (approximately 1.5 g each) were collected from the central portion of each dough, placed in 10 mm nuclear magnetic resonance (NMR) tubes, and tightly compressed to a height of 10.5 mm. The tubes were sealed with parafilm to prevent moisture loss and stored in a thermostatic cell at 25.0 ± 0.1 °C before analysis.

Free induction decay signals were acquired using a single 90° pulse, a dwell time of 7 μs, and a recycle delay of 0.9 s within a 0.5 ms acquisition window (the experimental limit ensuring magnetic field homogeneity), with 32 scans and 900 data points. Quantitative information on proton relaxation times and the relative proportions of rigid and mobile proton populations measurable within the FID time frame (7–500 μs) was obtained by fitting the decay curves to a two‐component model (exponential and Gaussian) using SigmaPlot v.6 (Systat Software Inc., Chicago, United States of America USA), using the following equation:

(4) $$f\left(t\right)=y_{0}+ae^{\left(-\frac{t}{T_{A}}\right)}+ce^{{\left(-\frac{t}{T_{B}}\right)}^{2}}$$

where *y*_0_ is the intercept, *a* and *c* are the relative abundances (%) of populations *A* and *B*; *T*_*A*_ and *T*_*B*_ are the relaxation times (μs) of the relative populations (*T*_*A*_ and *T*_*B*_).

Proton transverse relaxation time (¹H *T*₂) was measured using the CPMG pulse sequence with a recycle delay of 1 s, an interpulse spacing of 0.04 ms, 2500 data points, and 32 scans. The resulting ¹H *T*₂ curves were analyzed as quasi‐continuous relaxation time distributions using UPEN software (Alma Mater Studiorum, Bologna, Italy). In addition, ¹H *T*₂ CPMG relaxation decays were fitted to a discrete exponential model using SigmaPlot v.6 (Systat Software Inc.) to determine relaxation times and proton population abundances using the following equation:

(5) $$f\left(x\right)=y_{0}+ae^{-\mathrm{bx}}+ce^{-\mathrm{dx}}+ge^{-\mathrm{hx}}+ie^{-\mathrm{fx}}$$

where *y*_0_ is the intercept, *a*, *c*, *g* and *i*, the relative abundances (%) of population *C, D, E*, and *F*, and *b*, *d*, *h*, and f the relaxation times (ms) of population C, D, E and F, respectively (*T*_2C_, *T*_2D_, *T*_2E_, *T*_2F_).

Five FID and five CPMG curves were acquired for each sample.

### Statistical analysis

Means and standard deviations were calculated using SPSS (version 27.0, SPSS Inc., Chicago, IL, USA) statistical software. Two‐way analysis of variance (ANOVA) was performed by using the same software to assess significant differences (*P* < 0.05) due to the type of flour (*F*) and the type of antioxidant (*A*) in cracker formulations, and their interaction (*F* × *A*). Tukey HSD analysis was used as a post‐hoc test.

## RESULTS AND DISCUSSION

### Physico‐chemical properties of crackers

#### Peroxide value and total antioxidant capacity of cracker samples

**Figure 1:**
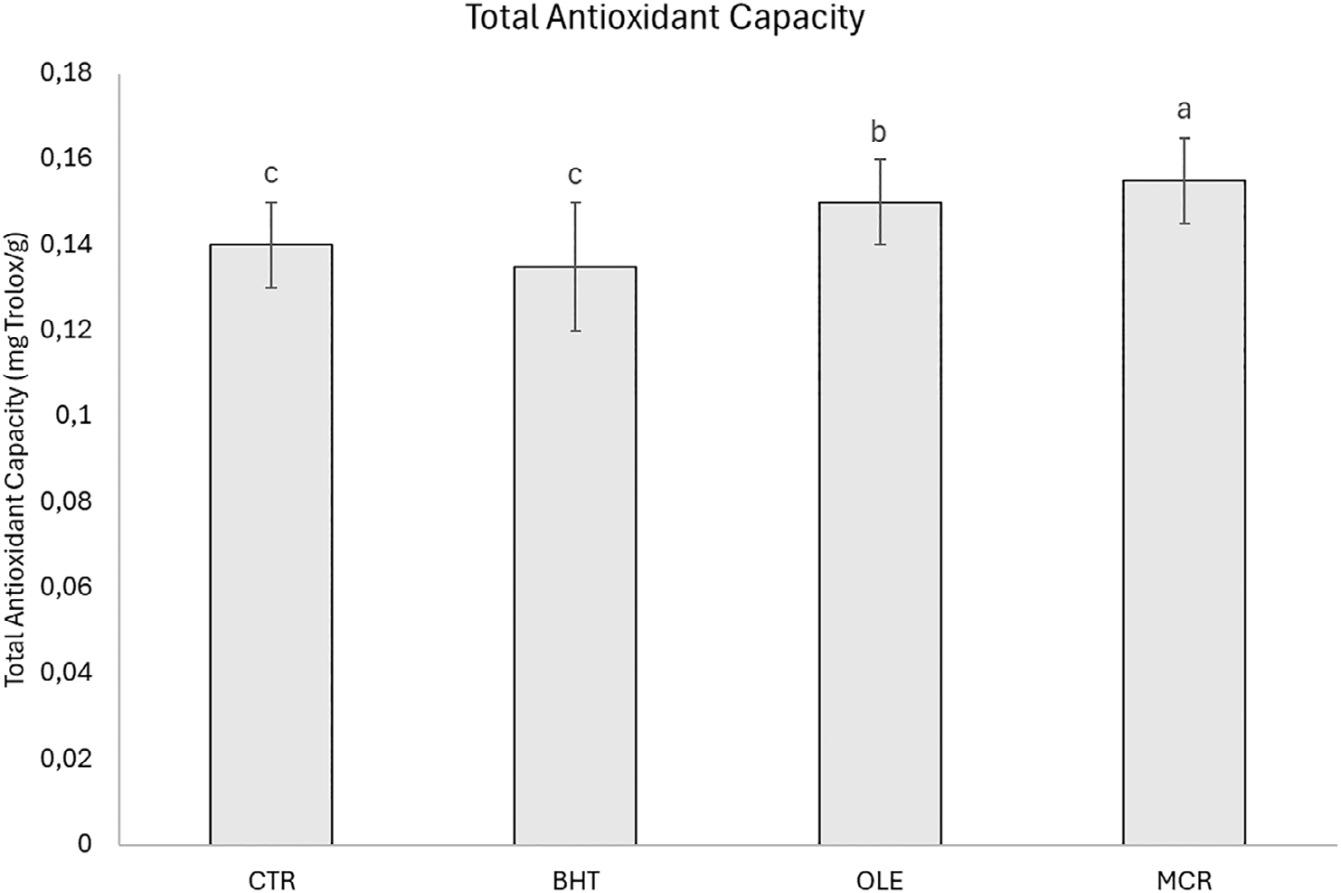
Total antioxidant capacity of cracker samples as a function of antioxidant type. Data are reported as means ± standard deviations (*n* = 12). Different small letters indicate significant differences (*P* < 0.05) among cracker samples as a function of antioxidant type. Abbreviations of cracker samples: CTR, control samples without the addition of antioxidants (RF‐CTR and WF‐CTR); BHT, samples with synthetic antioxidant (BHT) (RF‐BHT and WF‐BHT); OLE, samples with olive leaf extract (OLE) in a free form (RF‐OLE and WF‐OLE); MCR, samples with olive leaf extract (OLE) in an encapsulated form (RF‐MCR and WF‐MCR).

After molding/shaping and cooking the crackers, the effects of the different formulations and antioxidant additions on the peroxide value and total antioxidant capacity were evaluated (Table 3, Fig. 1). A significant effect of the used antioxidant (*A*) on peroxide value, and a significant effect of both *A* and the interaction between flour and antioxidant (*F* × *A*) on total antioxidant capacity were observed. A significant effect of factor *A* (*P* < 0.001) on peroxide value indicated that samples containing OLE, either in free or encapsulated form, remained below the limit of 20 meq O₂ kg⁻¹ fat, whereas CTR and RF–BHT samples exceeded this threshold. The CTR samples showed the highest peroxide values, and the OLE samples showed the lowest. The BHT and MCR samples did not differ significantly from either CTR or OLE samples. These findings demonstrate that the addition of free OLE reduced the peroxide value significantly, whereas encapsulated OLE and BHT did not produce a significant change immediately after production. Similarly, Paciulli *et al*.^19^ reported that adding either free or encapsulated OLE did not affect the peroxide value of biscuits significantly at the time of production. No significant effects of factor *F* or the *F* × *A* interaction were observed. Wholemeal flour contains more lipids than refined flour but it also provides higher antioxidant content, which exerts a protective effect on lipids.^22^ Overall, peroxide value analysis confirmed that free OLE induced a significant immediate reduction after cracker production, whereas neither BHT nor encapsulated OLE produced a comparable effect.

**Table 3:** Peroxide value, total antioxidant capacity, moisture content, texture (peak positive force, area) and color parameters (L*, a*, b*, ***ΔE***) of cracker samples

Sample	Peroxide value (mEq O_2_/kg fat)	Total antioxidant capacity (mg Trolox/g)	Moisture content (g kg^−1^)	Texture parameters		Color parameters			
				Peak positive force (N)	Area (N s)	L*	a*	b*	*ΔE*
RF‐CTR	22.36 ± 6.16^ax^	0.14 ± 0.01^cx^	44.0 ± 6.5^abx^	13.66 ± 3.81^ax^	8.72 ± 3.01^abx^	85.86 ± 10.05^ax^	1.48 ± 0.60^bx^	28.65 ± 3.28^ax^	–
WF‐CTR	22.80 ± 3.99^ax^	0.14 ± 0.01^cy^	27.5 ± 4.9^aby^	6.45 ± 1.28^ay^	4.40 ± 1.20^aby^	62.66 ± 6.74^ay^	10.72 ± 2.01^by^	26.78 ± 2.93^ay^	–
RF‐BHT	20.10 ± 4.08^abx^	0.13 ± 0.02^cx^	33.8 ± 15.9^bx^	13.48 ± 4.20^ax^	8.48 ± 3.46^bx^	79.05 ± 1.58^cx^	1.16 ± 0.48^bx^	26.39 ± 1.78^bx^	7.17^cd^
WF‐BHT	17.20 ± 2.24^abx^	0.14 ± 0.01^cx^	17.7 ± 8.0^by^	6.59 ± 2.18^ay^	4.30 ± 1.10^by^	53.62 ± 4.99^cy^	11.26 ± 1.26^by^	25.86 ± 1.01^by^	9.27^bc^
RF‐OLE	14.38 ± 2.66^bx^	0.16 ± 0.01^bx^	49.5 ± 26.3^abx^	15.85 ± 5.11^ax^	8.99 ± 4.47^abx^	76.72 ± 2.44^dx^	2.12 ± 0.78^ax^	29.26 ± 1.91^ax^	9.48^b^
WF‐OLE	15.72 ± 2.33^bx^	0.14 ± 0.01^bx^	14.6 ± 4.7^aby^	7.05 ± 1.78^ay^	4.08 ± 0.81^aby^	49.12 ± 2.75^dy^	12.20 ± 0.56^ay^	25.82 ± 1.19^ay^	13.70^a^
RF‐MCR	18.59 ± 4.83^abx^	0.16 ± 0.01^ax^	53.7 ± 18.9^ax^	13.06 ± 4.63^ax^	11.17 ± 4.27^ax^	79.82 ± 4.24^bx^	1.85 ± 0.55^bx^	29.35 ± 2.45^ax^	7.66^bcd^
WF‐MCR	17.44 ± 2.31^abx^	0.15 ± 0.01^ax^	24.2 ± 10.0^ay^	7.57 ± 2.56^ay^	4.54 ± 0.71^ay^	60.26 ± 5.55^by^	10.62 ± 1.09^by^	27.27 ± 1.60^ay^	5.98^d^
p F	n.s.	n.s.	***	***	***	***	***	***	n.d.
p A	***	***	*	n.s.	*	***	***	***	*
p F*A	n.s.	***	n.s.	n.s.	n.s.	**	**	**	n.d.

*Note*: Data are expressed as means ± standard deviations. Cracker samples: RF‐CTR, refined wheat flour cracker without the addition of antioxidant; WF‐CTR, whole wheat flour cracker without the addition of antioxidant; RF‐BHT, refined wheat flour cracker with the addition of BHT; WF‐BHT, whole wheat flour cracker with the addition of BHT; RF‐OLE, refined wheat flour cracker with the addition of OLE in a free form; WF‐OLE, whole wheat flour cracker with the addition of OLE in a free form; RF‐MCR, refined wheat flour cracker with the addition of encapsulated OLE; WF‐MCR, whole wheat flour cracker with the addition of encapsulated OLE. p F, p A, and p *F* × *A* refer to the effect of flour type, the type of antioxidant and their interaction, respectively. *, **, *** indicate significant differences at *P* < 0.05, *P* < 0.01, *P* < 0.001, respectively; ‘n.s.’ indicates no significant differences at *P* < 0.05, ‘n.d.’ means not determined. Means in a column with different superscripts are significantly different (*P* < 0.05); specifically, ‘x’, ‘y’ refer to the main effect of flour, and ‘a’, ‘b’, ‘c’ refer to the main effect of the type of antioxidant.

Although total antioxidant capacity showed only minor differences among cracker formulations, the significant effect of factor *A* (*P* < 0.001) indicated that MCR samples had the highest values, followed by OLE samples, while CTR and BHT samples exhibited similar, statistically indistinguishable values (Fig. 1). Similarly, Difonzo *et al*.,^17^ Conte *et al*.,^18^ and Caponio *et al*.^37^ observed that the addition of olive oil by‐products enhanced the antioxidant activity significantly in comparison with the CTR samples. The data also demonstrated that, although the differences in total antioxidant capacity among cracker samples were relatively small, encapsulation allowed this cracker sample parameter to be increased significantly immediately after the production process, more than OLE in a free form, indicating a protective effect of encapsulation on the bioactive compounds. The significant *F* × *A* interaction showed that the extent of the differences in total antioxidant capacity values in RF samples was higher than that observed in WF samples. The absence of a significant effect of *F* on the total antioxidant capacity of crackers may be associated to the presence of bound antioxidants, which were not quantified in the extraction conditions adopted. The contrasting effects of free and encapsulated OLE can be attributed to their release and stability during processing. Free OLE was immediately available to quench lipid radicals, reducing PV at production; however, the phenolic compounds were more prone to thermal degradation.^38^ On the other hand, encapsulation limited early antioxidant activity but protected phenolics from degradation, resulting in higher total antioxidant capacity values.^19^, ^39^ This suggests a balance between immediate oxidative protection with free OLE and long‐term preservation of antioxidant potential with encapsulation.

#### Moisture content, texture and color parameters of cracker samples

The physical properties of crackers are very important; a crisp, fragile, and crunchy texture is associated with high quality.^40^, ^41^, ^42^ The independent variables *F* and *A* and their interaction *F* × *A* had significant effects on the moisture content and color of the samples (Table 3).

Considering the moisture content, values of both RF and WF cracker samples were within the commercial range (moisture content < 70 ± 20 g kg⁻¹ wwb)^43^. Data showed a significant main effect of both *F* (*P* < 0.001) and *A* (*P* < 0.05), whereas their interaction, *F* × *A*, was not significant. The RF samples exhibited a significantly higher moisture content than the WF samples. This can be attributed to the shorter cooking time of RF compared to WF, as reported in the materials and methods section.

Considering the effect of *A*, CTR and OLE samples showed moisture content values that were not significantly different from BHT and MCR samples. However, MCR samples exhibited a significantly higher moisture content than BHT samples. Similar results were reported by Paciulli *et al*.,^19^ who found no significant differences in moisture content between OLE and encapsulated OLE biscuits compared to the CTR sample, suggesting that the addition of OLE, either in free or encapsulated form, did not affect this parameter. Conversely, Conte *et al*.^18^ reported a significantly higher moisture content in gluten‐free breadsticks enriched with olive leaf extract and olive mill wastewater. This increase rose with higher levels of added antioxidants and was associated with the fiber content of the natural antioxidants, which can retain water.^18^

Regarding texture analysis, a significant main effect of *F* (*P* < 0.001) on peak positive force was observed, whereas *A* and the *F* × *A* interaction did not significantly affect this parameter. Although RF samples had a shorter cooking time than WF samples, they exhibited a peak positive force approximately 50% higher than WF samples. This result is consistent with Cankurtaran Kömürcü,^44^ who reported that crackers produced with refined wheat flour had the highest hardness values in comparison with whole grain crackers. The authors attributed this finding to the different composition of refined and whole grain crackers; the latter contain wheat bran fractions and lower gluten content than refined samples, which could explain the lower hardness values that were observed.^44^

The area under the force versus time curve, a parameter associated with the energy required to penetrate the sample, showed a significant main effect of *F* (*P* < 0.001) and *A* (*P* < 0.05), whereas the interaction *F* × *A* did not significantly affect this parameter. The results were consistent with those observed for peak positive force. According to Cankurtaran Kömürcü,^44^ this finding can be attributed to the different composition of flours. Considering the effect of *A*, the CTR and OLE samples did not differ significantly from the other cracker formulations. The MCR samples exhibited a higher area only compared to BHT‐OLE samples, and similar results were reported by Paciulli *et al*.^19^ in biscuits formulated with OLE. Similarly, Caponio *et al*.^37^ found comparable hardness values in CTR gluten‐free breadsticks and breadsticks enriched with olive cake. Conversely, Conte *et al*.^18^ observed significantly lower hardness in OLE‐enriched breadsticks in comparison with CTR samples and attributed this result to the higher moisture content of OLE samples.

The color parameters L*, a*, and b* were significantly influenced by *F, A*, and their interaction. The WF samples exhibited significantly lower lightness (L*) and yellowness (b*), and higher redness (a*), compared to the RF samples (*P* < 0.001). Considering the effect of *A* (*P* < 0.001), CTR samples showed the highest lightness (L*), followed by MCR and BHT samples, with OLE samples showing the lowest value. The OLE samples exhibited the highest redness (a*), whereas BHT samples had the lowest yellowness (b*); all other samples did not differ significantly for these parameters.

A decreased lightness (lower *L*⁎) and increased redness and yellowness (higher *a*⁎ and *b*⁎) when using whole instead of refined wheat flours in bakery products has been reported in the literature,^20^, ^45^, ^46^ and similar results on cereal snacks were reported by Cankurtaran Kömürcü.^44^ The data in the current study were consistent with these findings, except for yellowness, the decrease in which in WF crackers could be attributed to the specific product formulation and processing conditions. The longer cooking time of WF in comparison with the RF samples may also have influenced these results.

Consistently with the literature,^19^, ^37^ encapsulation significantly reduced the impact of OLE on the lightness (L*), and did not affect the redness (a*) and yellowness (b*) of crackers. Conversely, the presence of free OLE significantly increased the redness (*a*⁎), in agreement with results reported by Paciulli *et al*.^19^ and Caponio *et al*.^37^ in OLE and encapsulated OLE biscuits, and in breadsticks enriched with olive cake, respectively. In contrast, Conte *et al*.^18^ observed a significant decrease in redness in OLE‐enriched breadsticks compared to CTR. The addition of OLE, whether free or encapsulated, did not affect yellowness (*b*⁎), consistent with Conte *et al*.,^18^ whereas Paciulli *et al*.^19^ reported a significant increase in both OLE and encapsulated OLE biscuits, and Caponio *et al*.^37^ found a significant reduction in breadsticks enriched with olive cake. This variability in color results may be attributed to the type and amount of antioxidant used, the formulation ingredients, and the processing conditions.

The *Δ*E values were determined separately for RF and WF samples for each antioxidant type, in comparison with the respective CTR samples (Table 3). A significant main effect of *A* was observed (*P* < 0.05). The *Δ*E values of all samples exceeded 5, indicating that the color differences between antioxidant‐enriched and control samples were clearly perceptible to the human eye, and that all antioxidant‐enriched samples exhibited distinct colors in comparison with the controls (Table 3).^47^

Overall, the results indicated that encapsulation mitigated the impact of OLE on the color of both refined and whole wheat flour crackers. The RF‐MCR samples did not differ significantly from RF‐BHT, while in whole wheat formulations, the WF‐MCR samples were more comparable with WF‐CTR than WF‐BHT. In contrast, the addition of free OLE increased the *Δ*E values in both refined and whole wheat crackers. BHT had a smaller effect on cracker color than free OLE, highlighting an advantage of synthetic antioxidants. Nevertheless, encapsulated OLE minimized color deviations relative to CTR samples, achieving performance equal to or superior to BHT, with whole wheat flour further enhancing this effect.

### Molecular properties of cracker dough samples

#### Attributes of ^1^H NMR populations

The mobility of water and biopolymers, and the distribution of water molecules among different domains at molecular level, can be associated with the physical properties and quality of cereal‐based dough/batter and final products.^48^ Table 4 reports the ^1^H populations’ relaxation times and abundances for all samples.

**Figure 2:**
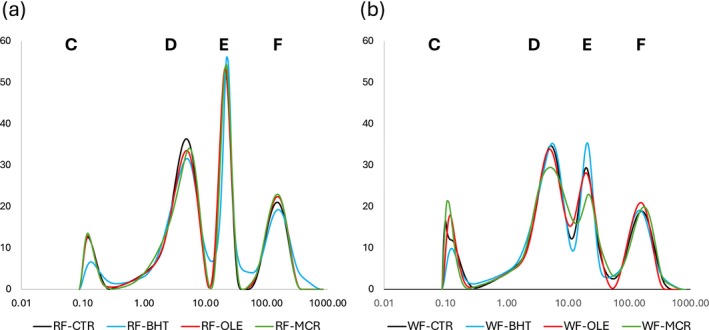
Representative Carr–Purcell–Meiboom–Gill (CPMG) ^1^H T_2_ distributions of cracker doughs reported as a function of flour refinement degree and antioxidant type. (a) Comparison among cracker doughs formulated with refined wheat flour: control sample (RF‐CTR, black lines), BHT sample (RF‐BHT, light blue lines), OLE sample (RF‐OLE, orange lines), MCR sample (RF‐MCR, green lines). (b) Comparison among cracker doughs formulated with whole wheat flour: control sample (WF‐CTR, black lines), BHT sample (WF‐BHT, light blue lines), OLE sample (WF‐OLE, orange lines), MCR sample (WF‐MCR, green lines).

The FID experiment showed the presence of two proton populations named A (the less mobile one) and B (the more mobile one), relaxing in the range of 14.57–16.47 μs and 324.43–366.60 μs, respectively (Table 4). The ^1^H T_2_ distributions of the relaxation times showed the presence of four populations identified as popC, popD, popE, and popF, from the least to the most mobile proton population, respectively. Figure 2 shows representative CPMG ^1^H T_2_ distributions of dough samples as a function of the independent variables, *F* and *A*. The ^1^H relaxation times were in the range of 0.33–0.70 ms, 3.83–4.28 ms, 15.11–20.16 ms, and 99.01–129.97 ms, for populations C, D, E and F, respectively. Considering the abundances of the ^1^H populations, the relative abundance of population A + population B gave 100% of the FID proton signal, whereas the relative abundance of populations C, D, E, and F gave 100% of the CPMG signal. The dominant FID population was population A, which represented 78.23% to 82.62% of the total observable protons. In the ^1^H T_2_ timeframe, the dominant population was population D, representing 34.86% to 38.86% of the total detectable protons, followed by population E (28.46% to 33.02%), population F (21.31% to 26.36%), and population C (8.22% to 10.65%). The relaxation times of populations B and C overlapped; these proton populations were therefore considered to represent the same protons, and only population C was considered as belonging to the better resolved CPMG experiment signal. As further confirmation of this hypothesis, the relaxation time of populations B and C showed the same results as a function of the tested variables (data not shown).

**Table 4:** Proton nuclear magnetic resonance (^1^H NMR) results as a function of flour and antioxidant types in cracker doughs

Sample	popA (%)	popC (%)	popD (%)	popE (%)	popF (%)	T_A_ (μs)	T_2C_ (ms)	T_2D_ (ms)	T_2E_ (ms)	T_2F_ (ms)
RF‐CTR	82.17 ± 0.34^ax^	8.99 ± 0.43^ax^	37.47 ± 0.22^ax^	32.03 ± 0.26^abx^	21.52 ± 0.36^bx^	14.86 ± 0.20^bx^	0.53 ± 0.06^ax^	4.00 ± 0.12^ax^	17.95 ± 0.46^bx^	115.53 ± 4.93^ax^
WF‐CTR	79.43 ± 0.24^ay^	10.27 ± 0.13^ay^	36.78 ± 0.22^ay^	30.11 ± 0.48^aby^	22.85 ± 0.60^bx^	16.13 ± 0.10^by^	0.42 ± 0.05^ay^	4.05 ± 0.17^ax^	16.10 ± 0.74^by^	110.41 ± 5.54^ax^
RF‐BHT	81.64 ± 0.25^abx^	9.16 ± 0.52^ax^	36.45 ± 1.53^ax^	32.00 ± 1.47^abx^	24.07 ± 1.80^abx^	14.81 ± 0.12^abx^	0.60 ± 0.03^ax^	4.13 ± 0.07^ax^	19.41 ± 0.81^ax^	118.70 ± 11.87^ax^
WF‐BHT	79.39 ± 0.24^aby^	10.06 ± 0.14^ay^	37.98 ± 0.63^ay^	29.92 ± 1.17^aby^	22.04 ± 0.59^abx^	15.89 ± 0.09^aby^	0.44 ± 0.07^ay^	4.08 ± 0.07^ax^	17.02 ± 0.45^ay^	116.36 ± 3.50^ax^
RF‐OLE	81.96 ± 0.46^abx^	8.81 ± 0.46^ax^	35.27 ± 0.22^bx^	32.64 ± 0.36^ax^	23.28 ± 0.34^abx^	14.87 ± 0.12^abx^	0.54 ± 0.03^ax^	3.99 ± 0.10^ax^	18.30 ± 0.35^bx^	115.65 ± 2.59^ax^
WF‐OLE	79.09 ± 0.58^aby^	10.02 ± 0.16^ay^	36.28 ± 0.19^by^	30.95 ± 0.27^ay^	22.75 ± 0.24^abx^	16.25 ± 0.06^aby^	0.43 ± 0.06^ay^	4.08 ± 0.12^ax^	16.18 ± 0.34^by^	111.63 ± 1.92^ax^
RF‐MCR	81.44 ± 0.47^bx^	9.12 ± 0.32^ax^	35.36 ± 0.11^bx^	31.86 ± 0.57^bx^	23.66 ± 0.56^ax^	15.00 ± 0.06^ax^	0.62 ± 0.05^ax^	4.11 ± 0.10^ax^	18.59 ± 0.36^bx^	115.31 ± 3.90^ax^
WF‐MCR	78.82 ± 0.59^by^	10.24 ± 0.30^ay^	36.13 ± 0.36^by^	29.47 ± 0.72^by^	24.17 ± 0.70^ax^	16.32 ± 0.12^ay^	0.41 ± 0.06^ay^	3.98 ± 0.06^ax^	15.89 ± 0.53^by^	112.61 ± 10.67^ax^
*p F*	***	***	**	***	n.s.	***	***	n.s.	***	n.s.
*p A*	**	n.s.	***	*	**	***	n.s.	n.s.	***	n.s.
*p F* × *A*	n.s.	n.s.	*	n.s.	**	*	n.s.	n.s.	n.s.	n.s.

*Note*: Data are expressed as means ± standard deviations. Cracker samples: RF‐CTR, refined wheat flour cracker without the addition of antioxidant; WF‐CT, whole wheat flour cracker without the addition of antioxidant; RF‐BHT, refined wheat flour cracker with the addition of BHT; WF‐BHT, whole wheat flour cracker with the addition of BHT; RF‐OLE, refined wheat flour cracker with the addition of OLE in a free form; WF‐OLE, whole wheat flour cracker with the addition of OLE in a free form; RF‐MCR, refined wheat flour cracker with the addition of encapsulated OLE; WF‐MCR, whole wheat flour cracker with the addition of encapsulated OLE. p F, p A, and p *F* × *A* refer to the effect of flour type, the type of antioxidant and their interaction, respectively. *, **, *** indicate significant differences at *P* < 0.05, *P* < 0.01, *P* < 0.001, respectively; ‘n.s.’ indicates no significant differences at *P* < 0.05. Means in a column with different superscripts are significantly different (*P* < 0.05); specifically, ‘x’, ‘y’ refer to the main effect of flour, and ‘a’, ‘b’, ‘c’ refer to the main effect of the type of antioxidant.

The above results were consistent with the literature on wheat flour dough^49^, ^50^, ^51^, ^52^, ^53^ and wheat flour biscuit dough^54^ reporting the presence of two ^1^H FID and four ^1^H T_2_ populations. On the other hand, Li *et al*.,^2^ Li *et al*.,^23^ and Assifoui *et al*.^55^, ^56^ reported the presence of three CPMG populations in WF cracker and biscuit dough, respectively. Different experimental conditions related to the product but also to the algorithms used for data processing could account for the discrepancies in the number of populations found by different research groups.

As far as the authors are aware, only limited information about the proton molecular mobility and dynamics of a wheat flour cracker dough has been reported in the literature – by Li *et al*.^2^ and Li *et al*.^23^ However, those studies focused on cracker dough formulations with ingredients and proportions differing from those used in the present work. Other authors investigated the molecular properties of biscuit doughs which were produced adopting a formulation with similar amount of lipids but also containing additional ingredients in a different amount compared to those used in cracker dough.^54^, ^55^, ^56^ In the present study, the proton attributions were performed considering the above studies, together with the scientific literature about refined and bran‐enriched wheat flour doughs.^51^, ^52^, ^57^, ^58^ According to Bosmans *et al*.,^52^ Assifaoui *et al*.,^55^, ^56^ and Serial *et al*.,^54^ population A can be assigned to protons in solid‐like components, as starch, representing the main biopolymers in both refined and unrefined wheat flour, but also to gluten proteins, and water molecules closely associated with those of solids. Population E, relaxing in the time range of 10–100 ms, was reported to be the dominant population in the CPMG proton distribution of wheat flour doughs.^23^, ^48^, ^51^, ^52^, ^54^, ^57^, ^58^

In the present study, population E was the second most abundant proton population after population D. In previous research, the assignment of population D has varied slightly. In wheat flour dough, population D has been attributed to OH protons of intragranular water and starch, together with CH protons of gluten and exchanging protons of confined water and gluten.^52^ In contrast, in wheat flour crackers it has been related to overlapping signals from extra‐granular starch water and water in the gluten matrix.^2^

The second most abundant proton population, popE, was assigned to mobile protons of water in exchange with OH protons of extra‐granular starch and exchanging protons of gluten.^52^ Population C was assigned to CH protons of amorphous starch and gluten in limited contact with water in wheat flour dough,^52^ and to dietary fiber constituents such as arabinoxylan, as previously reported in wheat flour dough enriched with bran.^2^, ^51^, ^57^ Population F, the most mobile proton population, has been variably assigned to lipids, weakly bound water, or both in the literature.

In the study by Hemdane *et al*.,^57^ investigating proton mobility and dynamics in wheat flour doughs and wheat bran fractions, the most mobile proton population, with a relaxation time of approximately 100 ms, was assigned to lipids, as its relaxation time was similar to that of isolated germ oil. Assifoui *et al*.^55^, ^56^ assigned slowly relaxing protons (with a relaxation time of approximately 105 ms) to apolar protons corresponding to the fat fraction in biscuit dough. Conversely, Li *et al*.,^2^ Li *et al*.,^59^ and Wang *et al*.^53^ investigating refined and unrefined/bran‐enriched wheat flour doughs, assigned this proton population, with relaxation times in the ranges 100–300 ms, 30–100 ms, and 37–115 ms, respectively, to weakly bound protons of water. Parenti *et al*.^58^ assigned the most mobile proton population in whole wheat flour dough, with a relaxation time of 41.84–53.76 ms, to both weakly bound protons of water and lipids. In the present study, considering the formulation of wheat flour cracker dough (Table 4), it was hypothesized that popF could be assigned to both the protons of extra virgin olive oil (240 g kg⁻¹ of flour) in the apolar phase and to weakly bound (free) protons of water in the cracker dough system.

The flour type and addition of antioxidant in cracker dough affected ^1^H molecular mobility and dynamics (Table 4, Fig. 2). The differences obtained as a function of both independent variables were statistically significant (*P* < 0.05) but relatively small and so only their main effects are discussed in the following paragraphs.

#### The effect of the antioxidant type

The type of antioxidant showed a significant (*P* < 0.05) main effect on the following parameters: popA, popD, popE, popF, T_A_ and T_2E_ (Table 4). Despite the statistical significance of the data, it can be observed that the differences among samples were relatively small, suggesting that the addition of antioxidants had little impact on the proton molecular mobility and dynamics of cracker doughs. The relative abundances of proton populations and relaxation times of BHT and OLE doughs were the most similar to the CTR sample, except for the T_2E_ and popD results.

Little information is available in the literature regarding the effect of antioxidants on ¹H NMR mobility and dynamics in dough systems.^60^, ^61^ The present results showed that BHT significantly affected only *T₂E*, causing a marked increase in this parameter compared with the CTR sample. Consistent with the findings of Zheng *et al*.,^60^ who investigated tea polyphenols in non‐fermented frozen dough, it can be hypothesized that BHT introduced a small number of hydroxyl groups that promoted water binding on the surface or within the gluten network, thereby increasing the mobility of this proton population.

Considering the effect of OLE, the relative amount of popD was lower in OLE cracker dough than in the CTR dough. Zheng *et al*.^60^ reported a decrease and an increase in the relative abundances of *T₂* proton populations when 5 g kg⁻¹ and 1–2 g kg⁻¹ of tea polyphenols, respectively, were added to a wheat dough system. However, both the quantity and the type of polyphenols influenced the ¹H NMR results significantly.^60^

Most of the statistical differences observed among the tested cracker doughs relative to the CTR sample were associated with MCR doughs, which showed significant variations in popA, popD, popF, and TA. The MCR doughs exhibited a significantly higher TA value and lower relative abundances of popA and popD compared with the CTR sample. However, because these differences were small, MCR likely had little or no effect on the phenomenon under investigation. A greater effect was found for popF, the relative abundance of which was significantly higher in MCR than in CTR dough. These results suggest that MCR doughs, compared with CTR dough, were characterized by a redistribution of protons – specifically, a decrease in tightly and moderately bound protons and an increase in weakly bound ones. These differences may be attributed to the composition of the MCR antioxidant (Table 2), particularly the type and amount of its ingredients, compared with the other cracker formulations containing OLE and pea proteins.

Overall, the BHT and OLE samples exhibited molecular properties most similar to those of the CTR samples, showing only minor but significant differences in the T_2E_ and popD parameters, respectively. The MCR samples affected popA, popD, popF, and T_A_ significantly, although the differences were small for all parameters except popF, which showed a marked increase relative to the CTR samples. These findings indicate that the addition of BHT and OLE to the cracker dough formulation had only a limited effect on water redistribution among the main wheat flour components, and that encapsulated OLE decreased tightly and moderately bound protons while increasing weakly bound ones.

#### The effect of the flour refinement degree

The degree of flour refinement had a significant effect on the ¹H NMR parameters of cracker dough (Table 4 and Fig. 2). The ^1^H T_2_ distributions were more clearly resolved in RF dough than in WF dough (Fig. 2). The influence of bran and germ addition to wheat flour dough has been investigated by Li *et al*.,^23^ Li *et al*.,^62^ Lu and Seetharaman,^63^ Serial *et al*.,^54^ and Hemdane *et al*.^51^, ^52^, ^53^, ^54^, ^55^, ^56^, ^57^ However, only Li *et al*.^23^ examined this effect in cracker dough, whereas the other studies focused on products such as bread and biscuit dough.

The decrease in proton population resolution and consequently the increased molecular heterogeneity observed in WF compared with RF samples contrasted with the findings of Hemdane *et al*.,^51^, ^52^, ^53^, ^54^, ^55^, ^56^, ^57^ who reported the opposite trend when comparing refined wheat flour with wheat bran fractions, and refined wheat flour dough with bran‐enriched dough. Hemdane *et al*.^57^ found that proton populations of bran constituents (i.e., water‐unextractable arabinoxylan, cellulose, wheat germ oil, and wheat bran fractions) were more clearly resolved than those of wheat flour. Similarly, Hemdane *et al*.,^51^ who examined refined wheat flour dough enriched with wheat bran fractions (150–200 g kg⁻¹ of flour), observed that the proton populations in wheat flour dough were broader and therefore less resolved than those in bran‐rich doughs.

This difference may be attributed to the distinct formulations of the cracker samples in this study compared with bread dough. Crackers contained a relatively lower water content and a higher amount of extra virgin olive oil than bread formulations.^51^ The presence of extra virgin olive oil may explain the higher relative abundance of popF in cracker dough compared with values reported for bread dough.^57,58^ The ingredients used in the cracker formulation (Table 2) could have interacted with flour constituents and influenced the ¹H NMR distribution pattern.

The degree of flour refinement had a significant main effect on popA, popC, popD, popE, T_A_, T_2C_, and T_2E_ (Table 4). Based on the FID results, WF samples exhibited a 3.3% reduction in popA and an 8.4% increase in T_A_ compared with RF samples.

The greatest impact of the degree of flour refinement on the relative abundances of proton populations was observed for popC and popE, which were significantly higher (12.3%) and lower (6.2%), respectively, in WF dough than RF dough. Smaller but significant differences were found for popD, which was 1.3% lower in WF cracker dough than in RF dough. Regarding the relaxation times of proton populations in WF samples, T_2C_ and T_2E_ were reduced significantly, by approximately 26.8% and 12.8%, respectively, relative to RF dough.

The above results are consistent with Li *et al*.,^23^ Li *et al*.^62^ and Hemdane *et al*.^51^, ^57^ Considering tightly bound protons from the FID experiment, Hemdane *et al*.^51^ observed that wheat flour bread dough enriched with bran fractions (coarse, ground, and pericarp enriched) exhibited significantly lower popA than wheat flour dough. The authors attributed the decrease in popA to the higher water content of bran‐rich doughs.^51^ In the current study, the dough water content was constant across samples. The lower popA in WF dough is therefore most likely related to the estimated lower starch content in WF in comparison with RF dough, as also reported by Bosmans *et al*.^52^

For the CPMG experiment, the results of the current study were consistent with Li *et al*.,^23^ Li *et al*.,^62^ and Hemdane *et al*.^57^ who observed a migration of water from gluten into the arabinoxylan matrix in WF cracker dough, WF bread dough, and wheat bran fractions versus refined wheat flour. Indeed, in the above studies the addition of wheat bran caused a significant increase of the relative abundance of protons assigned to the water absorbed by the arabinoxylan matrix and starch (relaxation time = 0–1 ms) and a significant decrease in protons attributed to water absorbed by the gluten network (relaxation time = 1–100 ms).^23^, ^57^, ^62^ Hemdane *et al*.^51^ found a similar increase in protons assigned to water absorbed by arabinoxylan matrix and starch but also a simultaneous increase in popE, a result most likely linked to the higher water content used by the authors in the bran‐rich dough (726–73.6 g kg^−1^ of flour) in comparison with control dough (603 g kg^−1^ of flour).

Concerning the relaxation times of proton populations, Lu and Seetharaman,^63^ consistent with the results of this study, observed a significant reduction in the mobility of more mobile protons (CPMG experiment) when refined wheat flour dough was enriched with whole grain barley flour. This outcome suggests that the addition of bran caused a significant decrease in proton mobility within the dough system.

Overall, the results revealed significant changes in proton mobility and dynamics in WF in comparison with RF dough. The WF dough exhibited significantly lower mobility of the more mobile protons, a higher proportion of protons were associated with the fiber fraction, and a lower proportion were associated with the gluten matrix.

## CONCLUSIONS

The present study expanded knowledge on the substitution of synthetic antioxidants with antioxidants from olive oil by‐products, and investigated the effect of using WF instead of RF flours in cracker formulations targeting clean‐label, wholemeal cereal‐based products.

The results demonstrated that encapsulation of OLE improved overall cracker quality. Although only free OLE reduced the peroxide value after processing, it showed intermediate total antioxidant capacity, higher than CTR and BHT but lower than encapsulated OLE, and had the greatest effect on lightness and redness among all cracker formulations. Encapsulated OLE, in contrast, maximized the total antioxidant capacity, whereas BHT did not increase this parameter.

As with BHT, encapsulated OLE did not change the moisture content and texture of cracker samples as compared to the control. The encapsulation was also able to mitigate the impact of antioxidants on crackers' color, showing a better performance than BHT. The ¹H NMR molecular mobility and dynamics of cracker doughs were minimally affected by antioxidants and were largely determined by flour refinement degree, indicating lower mobility in WF dough and redistribution of water among fibers and gluten proteins.

Overall, these findings highlight the potential of encapsulated OLE as a functional antioxidant in cereal‐based snacks, although its effect on oxidative stability during storage requires further investigation.

## AUTHOR CONTRIBUTIONS

Ottavia Parenti: conceptualization; data curation; formal analysis; investigation; writing – original draft; writing – review and editing. Maria Paciulli: conceptualization; data curation; formal analysis; investigation; project administration; supervision; writing – review and editing. Eleonora Carini: data curation; supervision; writing – review and editing. Francesca Bot: supervision; writing – review and editing. Emma Chiavaro: conceptualization; funding acquisition; project administration; supervision; writing – review and editing.

## FUNDING INFORMATION

This work was supported by the project funded under the National Recovery and Resilience Plan (NRRP), Mission 4 Component 2 Investment 1.3 ‐ Call for tender No. 341 of15 March 2022 of Italian Ministry of University and Research funded by the European Union – NextGenerationEU; Project code PE00000003, Concession Decree No. 1550 of 11 October 2022 adopted by the Italian Ministry of University and Research, Project title “ON Foods ‐ Research and innovation network on food and nutrition Sustainability, Safety and Security – Working ON Foods.

## CONFLICT OF INTEREST

The authors declare no conflict of interest.

## Data Availability

The authors declare that the data supporting the findings of this study are available within the paper. Should any raw data files be needed in another format they are available from the corresponding author upon reasonable request.
